# The context and potential of epigenetics in oncology

**DOI:** 10.1038/sj.bjc.6604930

**Published:** 2009-02-17

**Authors:** J Lopez, M Percharde, H M Coley, A Webb, T Crook

**Affiliations:** 1 The Breakthrough Toby Robins Breast Cancer Research Centre, The Institute of Cancer Research, 237 Fulham Road, London SW3 6JB, UK; 2 Faculty of Health and Medical Sciences, University of Surrey, Guildford, Surrey GU2 7XH, UK; 3 Department of Oncology, Royal Sussex University Hospital, Brighton, Sussex, UK

**Keywords:** epigenetics, methylation, oncology

## Abstract

Cancer has long been known to be a disease caused by alterations in the genetic blueprint of cells. In the past decade it has become evident that epigenetic processes have a function, at least equally important, in neoplasia. Epigenetics describes the mechanisms that result in heritable alterations in gene expression profiles without an accompanying change in DNA sequence. Genetics and epigenetics intricately interact in the pathogenesis of cancer (Esteller, 2007). In this review, we paint a broad picture of current understanding of epigenetic changes in cancer cells and reflect on the immense clinical potential of emerging knowledge of epigenetics in the diagnosis, prognostic assessment, treatment, and screening of cancer.

## Background

The most widely studied epigenetic modification is the cytosine methylation of DNA within the CpG dinucleotide (Jones and Baylin, 2007). The frequency of the CpG dinucleotide in the human genome is much less than expected, approximately 2–5%. CpG dinucleotides are not equally distributed throughout the genome; instead, they occur in clusters of either large repetitive sequences (such as rDNA, satellite sequences, or centromeric repeats) or in short CG-rich DNA stretches, known as ‘CpG islands’, found preferentially in the promoter region of genes. The majority of these ‘CpG islands’ are associated with housekeeping genes and typically occur close to the transcription start site of the gene. Such CpG islands are normally unmethylated, consistent with the ability of genes containing these islands within their promoter region to be transcribed in the presence of necessary transcription factors. Methylation of cytosine residues within CpG islands is associated with binding of methyl-binding domain proteins, recruitment of histone deacetylases (HDACs) and histone methyltransferases, histone modification, chromatin condensation, and transcriptional inactivation of the associated genes. The orchestration of methylation in CpG islands by an assortment of methylating and demethylating enzymes is thought to provide one of the layers of epigenetic control of germ-line and tissue-specific gene expression.

In contrast, repetitive genomic sequences that are scattered throughout the rest of the genome are heavily methylated and it is speculated that this may have played an important role over the course of evolution in maintaining the large amount of non-coding DNA in a transcriptionally inert state and also the silencing of endoparasitic and retroviral transposons.

Histones, the protein backbone of chromatin, are also important in epigenetics. Today, they are recognised as being important translators between genotypes and phenotypes, having a dynamic function in the regulation of chromatin structure and gene activity. Understanding the importance of histones in the normal cell and how this changes in neoplasia is still in its infancy compared with that of DNA methylation. Histones can be modified by acetylation, methylation, phosphorylation, sumoylation, and ubiquitination, all of which fine-tune the accessibility of DNA to transcription factors and the subsequent protein interactions that determine chromatin structure. Particular histone tail modifications have been directly linked to active or repressed transcription. For example, acetylation of certain N-terminal lysine residues by histone acetyltransferases (HAT) is associated with actively transcribed regions. The reverse process of histone deacetylation, catalysed by HDACs, results in a tighter closed formation of chromatin and suppression of DNA transcription. These are carefully conducted in conjunction with CpG island hypermethylation and provide an additional layer of epigenetic control. Recently, it has been suggested that histones are involved in establishing and maintaining ‘epigenetic memory’ (reviewed in Esteller, 2007).

DNA methylation and histone modifications are not isolated events, but occur in higher-order chromatin structure. We are only beginning to scratch the surface of understanding how nucleosome stability is modulated by the complex interplay between histone modifications and chromatin-binding proteins to fine-tune gene expression (reviewed in Berger, 2007). The most recently emerged participant in the epigenetic field is a family of small regulatory RNAs called microRNAs (miRNAs). The importance of miRNAs in orchestrating gene expression, primarily by incorporation into a silencing machinery termed the ‘RISC’ complex, is becoming evident. Within this complex, miRNAs bind to partially complementary target sites in the 3′-UTR of genes and can direct either translational inhibition or mRNA degradation. In this way, repression by miRNAs is one more way in which gene expression may be modulated outside changes to DNA sequence.

Methods for analysis of the epigenome have classically utilised bisulphite modification of genomic DNA. The ‘gold standard’ technique for assessment of individual CpG dinucleotides is bisulphite sequencing. A frequently used method for analysis of methylation, for example in DNA isolated from cancer tissues, is methylation-specific PCR. This technique uses PCR primers that discriminate between methylated and unmethylated DNA in bisulphite-modified genomic DNA. The methylation status of individual candidate gene CpG islands can readily be assessed this way, for example in cancer biopsies and biofluids. More recently developed methods use immunoprecipitation to purify methylated DNA, facilitating high-resolution whole-genome DNA methylation profiling (the DNA methylome) (Down *et al*, 2008).

## Cancer

### Alterations in methylation pattern in cancer

In cancer, the methylation landscape is profoundly distorted. Human tumours undergo a global overall loss of DNA methylation, but also acquire hypermethylation at specific promoters (reviewed in Chuang and Jones, 2007). The underlying mechanisms that cause these changes are unknown, but there is a suggestion that at least a subset of DNA methylation changes occur early in tumour development and may even initiate carcinogenesis.

**Figure 1:**
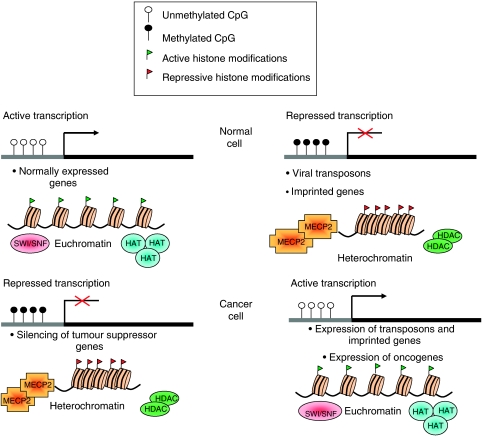
Current understanding of some of the changes to DNA and chromatin that occur in cancer cells. In the normal cell, promoters of actively transcribed genes are unmethylated and found within regions of euchromatin. Expression of other genes is repressed by promoter methylation and heterochromatin formation. In cancer, this is deregulated, resulting in the aberrant expression of normally silent genes and repression of tumour suppressor genes. Abbreviations: HAT, histone acetyltransferase; SWI/SNF, switch/sucrose nonfermentable nucleosome remodelling complex; MeCP2, methyl CpG-binding protein 2; HDAC, histone deacetylase.

Two consequences of losses of methylation have been proposed. First, weakening of transcriptional repression in normally silent regions of the genome could cause the potentially harmful expression of silenced genes, for example those imprinted; but also inserted viral and parasitic transposons. Second, global demethylation of the cell also affects chromosome stability. This is exemplified in patients with the rare condition of immunodeficiency – centromeric instability syndrome (germ-line mutation of DNA methylation enzyme DNMT3b), and in the DNMT1 knockout mouse (Jackson-Grusby *et al*, 2001). The latter model revealed a fascinating interaction between hypomethylation and p53. Conditional deletion of DNMT1 resulted in p53-dependent cell death that could be partially rescued by mutational inactivation of p53. Further, it was shown that up to 10% of genes were aberrantly expressed in hypomethylated fibroblasts and that changes in gene expression (that included growth factor receptors and proteins involved in signal transduction among others) were cell-type specific. In an analysis of primary human colorectal carcinomas, genome-wide demethylation was shown to correlate closely with chromosomal instability, further strengthening the hypothesis that there is a direct link between these two factors and the progression of carcinogenesis (Rodriguez *et al*, 2006; Figure 1). An earlier study in prostate cancer suggested a mechanistic association between genome-wide DNA hypomethylation and alterations on chromosome 8 (Schulz *et al*, 2002).

Concordant with the hypomethylation events, gene-specific DNA hypermethylation has been shown to occur in all cancers (Esteller *et al*, 2001a). Aberrant CpG island methylation has, up to the present time, been most commonly assessed in genes already known to be involved in tumour development, especially in tumour samples that do not harbour genetic alterations of the gene. This candidate gene approach has identified aberrant methylation-mediated silencing of genes involved in most aspects of tumorigenesis and several studies have confirmed that methylation-associated silencing inactivates tumour suppressor genes as effectively as mutations and is one of the cancer predisposing ‘hits’ in Knudson's classical two-hit model of carcinogenesis (Garinis *et al*, 2002), an hypothesis originally proposed by Jones and Laird (1999). Other genes that have CpG islands in their promoter region and have been shown to be subject to aberrant hypermethylation include those whose protein products are involved in the cell cycle, DNA repair, apoptosis, cell adhesion, and angiogenesis. In addition to these examples, a large number of genes are aberrantly methylated in cancers, yet lack an obvious function relevant to tumorigenesis. For example, the MYOD1 CpG island is methylated in multiple tumour types. It is a question of obvious interest as to why such genes are targets for methylation. This issue has to some extent been resolved by studies from several groups showing that genes that become methylated in cancer cells are specifically packaged with nucleosomes containing histone H3 trimethylated on Lys27. This packaging is established in these (unmethylated) CpG island genes early in development and then maintained in differentiated cell types by the presence of an EZH2-containing polycomb complex. In embryonic cells, the polycomb complex mediates reversible repression of genes involved in differentiation, but in cancer cells, the presence of the polycomb complex causes recruitment of DNA methyltransferases. Methylation ensues, leading to permanent rather than reversible gene silencing (Ohm *et al*, 2007; Schlesinger *et al*, 2007; Widschwendter *et al*, 2007).

With the development of genome-wide techniques, additional novel genes that contribute to tumorigenesis are certain to be identified. A key question is, of course, why specific sequences are targeted for hypermethylation in a biological milieu that is subject to global hypomethylation. How are tumour-specific methylation profiles established? It seems clear that tumours with a particular phenotype as a result of hypermethylation would have a clonal selective advantage leading to their increased survival. However, given that methylation patterns tend to be non-random and tumour specific (Costello *et al*, 2000), are there key switches that trigger the cascade of abnormal methylation patterns? One suggestion is that the particular combination of oncogenic transcription factors and the epigenetic machinery target particular sequences – as implied by the association *in vitro* of DNMT and the fusion protein product of pro-myelocytic leukaemia protein-1 (PML-RAR) and MYC (Croce *et al*, 2002) but this has not yet been shown to be a general mechanism of action. Another possibility is that the ‘local’ hypermethylation of specific tumour suppressor genes is the result of the role played by the environment or nutritional status. This hypothesis arose from the observation that the most heavily methylated tumours are those that arise from the gastrointestinal tract, presumably due to exposure to external carcinogens. One must now ask if other external factors, for example smoking habits or diet, can cause particular hypermethylation changes to tumour suppressor genes. Studies in a mouse model of silica-induced lung cancer have revealed that epigenetic silencing of *p16*^*INK4a*^ is an early event in the progression of lung cancer (at the stage of moderate dysplasia) (Blanco *et al*, 2007). Further study of this area, attempting to show a direct causal relationship between chemical damage, hypermethylation, and commitment to a pathway leading to lung carcinogenesis would clearly be interesting.

The most likely candidates for ‘key epimutations’ that may commit cells to carcinogenesis are the DNA repair genes. For example, epigenetic silencing of *BRCA1* results in the inability of cells to repair double-stranded DNA breaks, thereby relying on a more error-prone pathway that may result in the accumulation of mutations, potentially leading to cancer. The *hMLH1* mismatch repair gene is another well-characterised system of DNA repair. Deficiencies of this system result in mutation rates 100-fold greater than normal cells. In 90% of sporadic cases of colorectal, endometrial, and gastric cancers, which exhibit microsatellite instability but without a germ-line mutation in *hMLH1*, the gene is epigenetically inactivated (Herman *et al*, 1998), implying that a single epigenetic event may unleash new mutator pathways.

### Histone modifications in cancer cells

We are still largely ignorant of the intricate workings of histone packaging and its dynamic modulation of chromatin, and how these are disrupted in cancer. Preliminary attempts at profiling histone modifications on a genome level in a range of cell lines suggest that cancer cells exhibit a loss of monoacetylated and trimethylated forms of histone H4 (Fraga *et al*, 2005). Much work is ongoing to try to decode the histone map as well as understand how the complex interplay between histone modifications and DNA methylation is dysregulated in cancer. However, a mouse model of carcinogenesis has shown a few fascinating insights about the working of histones. Histones are present early-on in tumorigenesis and accumulate (Fraga *et al*, 2004) – suggesting that they too may have a function in the progression/transformation of neoplasia. Given the function histones may have in maintaining stable epigenetic memory, unravelling the workings of this process, and elucidating how it could be reverted or re-programmed to its ‘normal’ setting may open up therapeutic avenues for the future.

### Dysregulation of miRNAs

Global analysis of miRNA expression levels in several cancer types has shown that many miRNAs are also deregulated and may act as tumour suppressors. For example, deletion of mir-16 in chronic lymphocytic leukaemia leads to upregulation of one of its targets, the anti-apoptotic oncogene, *BCL2* (Cimmino *et al*, 2005), whereas miRNA *Let-7a-2* normally targets *RAS* and is downregulated in lung cancer (Takamizawa *et al*, 2004). Hypermethylation of CpG islands associated with specific miRNAs has, therefore, been proposed as one of the mechanisms by which the miRNA is selectively downregulated in tumours. Furthermore, in cases where the miRNA is situated in the coding region of a gene, methylation may simultaneously suppress expression of both the protein-coding gene and also its embedded miRNA.

## Tanslational epigenomics

### Epigenomic profiles as cancer cell markers

Methylated genomic DNA has several properties that make it an attractive potential biomarker in oncology. First, hypermethylation of most genes is rarely found in healthy individuals, although some changes may occur with age and environmental stresses. As such, the majority of methylation changes detected in cancer cells are acquired during neoplastic development and, therefore, specific to cancer. Second, methylated DNA is chemically stable and assays to detect it are highly sensitive and increasingly user friendly. DNA methylation assays can be performed on small biopsy samples obtained during the routine diagnostic work-up of patients, on archived frozen or paraffin-embedded tissue, and on the soluble genomic DNA found in the peripheral blood and other biofluids of many cancer patients (Table 1).

The ideal, which would be extremely helpful in the diagnostic and prognostic assessment of patients, would be a validated, specific, and sensitive panel of methylation markers that identifies individual cancer types. Thus far, only best-guess selections of a panel of candidate genes have been used. With the development of genomic techniques and an expansion in our understanding of the normal and diseased ‘methylome’, the prospect of a diagnostic methylation signature for each subtype of cancer becomes a genuine possibility.

Tumour-cell-derived DNA present in ‘luminal’ secretions, for example in saliva, sputum, gastrointestinal fluids, ductal lavage fluid, bronchoalveolar fluid, and pleural fluid, will offer an alternative source for methylation analysis. For example, a methylation screening panel of five candidate genes has been proposed to distinguish between malignant mesothelioma, primary lung adenocarcinoma, and normal lung (Tsou *et al*, 2005). Such a resource would be very valuable for respiratory clinicians to differentiate between lung cancer subtypes, each with its own distinct treatment regimen, when biopsies are difficult to obtain or persistently yield very small amounts of tissue. A significant proportion of diagnostic biopsies, often harvested from sites of metastatic disease, are reported as ‘undifferentiated carcinoma of unknown origin’. Such cancers frequently evade even the most skilled histopathologist and rigorous immunocytochemical analysis often fails to pinpoint the primary site. In these difficult diagnostic situations, where prognosis and treatment may vary widely between possible cancer types, a definitive methylation signature would be extremely useful in guiding management. It may even be possible to obtain such a signature from peripheral blood. Interestingly, it has been suggested that hypomethylation of L11 LINE sequences is particularly prevalent in urothelial cancers and might have diagnostic utility in these cancers (Jürgens *et al*, 1996). In a large case–control study, it has now been shown by measurement of the degree of global methylation in genomic DNA (using peripheral blood leukocytes) that DNA hypomethylation is indeed associated with increased risk of developing bladder cancer (Moore *et al*, 2008). Furthermore, this association is independent of smoking and the other risk factors for urothelial neoplasia. Assessment of global hypomethylation could therefore be a useful biomarker of susceptibility to urothelial (and perhaps other) cancer types.

### Screening

Hypermethylation of tumour suppressor genes is readily detectable in pre-malignant lesions, consistent with the hypothesis that epigenetic change occurs early in neoplasia. For example, CpG island hypermethylation is seen in *p16*^*INK4a*^, *p14*^*ARF*^, and *MGMT* in colorectal adenomas (Judson *et al*, 2006); and *MLH1* aberrant methylation in atypical endometrial hyperplasia (Banno *et al*, 2006). It is, therefore, entirely conceivable that DNA methylation may come to play a role in early detection screening. In this respect, the presence of methylated genomic DNA in a number of biofluids, as noted above, offers the possibility of non-invasive molecular screening for many common malignancies (Table 1). The major challenge in developing this approach will be the difficulties in achieving the high degree of sensitivity and specificity required. This is illustrated in a study of detection and quantification in serum of mutations in the *APC* gene in patients with colorectal cancer (Diehl *et al*, 2005). In the first instance, detection of methylated DNA in serum may be particularly helpful in individuals with a high familial risk of cancer. A careful study of methylation patterns of a panel of 10 candidate genes in inherited and sporadic breast and colorectal cancers showed that hereditary cancers ‘mimic’ the DNA methylation patterns present in the sporadic tumours (Esteller *et al*, 2001b), suggesting the development of a validated screening profile for a particular cancer, for example breast cancer, that will have great utility in management of high-risk patients in family history clinics. Hand in hand with screening is the potential for guiding early treatment strategies. In cervical cancer, different methylation events have been linked to distinct stages of HPV-induced malignant transformation in cell lines (Henken *et al*, 2007). Thus, the early detection of methylation signatures in cervical smears not only allows diagnosis of pre-malignant lesions, but also offers the potential for epigenetic therapies to be used upfront to reverse/prevent transformation.

### Epigenomic profiles as markers of tumour prognosis

To supplement traditional cancer prognosis methodologies, such as the classical TNM system that assesses tumour size, lymph node involvement, and distant metastasis, methylation patterns are now emerging as potentially highly informative staging modalities. Several small studies have used cluster analysis to link methylation patterns to specific clinical parameters. As additional methylated genes are identified and their prognostic utility revealed, this is likely to be an active area in the coming years. It is entirely foreseeable that clinicians will be making decisions on adjuvant treatment based on specific methylation signatures that specifically assess the ‘metastatic’ potential of an early cancer. A number of studies attest to the utility and clinical applicability of this approach. For example, detection of methylated DNA from the MYOD1 CpG island is associated with reduced disease-free and overall survival in cervical cancer (Widschwendter *et al*, 2004). Similarly, detection of circulating methylated DNA in the serum of patients with melanoma has prognostic value (Mori *et al*, 2005).

The other area in which epigenetics has great clinical potential is in the early detection of cancer relapse in routine patient follow-up. Serial analysis of serum or biofluid DNA methylation patterns may one day enable the detection of early relapse before it manifests with clinical symptoms or on routine imaging, affording an extended window in which cure may still be achievable. One small prospective study has already shown that gene methylation (panel of 10 genes) in saliva is a promising biomarker for the follow-up and early detection of still curable relapses of head and neck squamous cell carcinoma patients (Righini *et al*, 2007).

### Pharmacoepigenomics: epigenomic profiles as a marker of response to chemotherapy

The most exciting prospect for an oncologist is that methylation profiling may have utility in predicting the sensitivity of individual cancers to anti-cancer agents. The most compelling evidence for this is provided by the methylation-associated silencing of the DNA repair protein MGMT in glioblastomas. Hypermethylation of *MGMT* is the best independent predictor of response to temozolomide in gliomas (Hegi *et al*, 2005). Another example of a gene whose methylation-associated silencing is associated with relative drug resistance is the DNA mismatch repair gene *hMLH1*. Loss of *hMLH1* is associated with increased resistance to platinum compounds *in vitro* (Strathdee *et al*, 1999) and acquired methylation (detectable in peripheral blood) during treatment predicts poorer outcome following chemotherapy for ovarian cancer (Gifford *et al*, 2004).

### Therapeutic targets

Recently, increasing evidence supports the hypothesis that acquired resistance to chemotherapy results from progressive accumulation of epigenetic changes (Glasspool *et al*, 2006) and this inevitably leads to the question of whether these changes are reversible. Studies *in vitro* and in model systems certainly suggest that treatment with demethylating agents can reverse drug resistance (Plumb *et al*, 2000). These and other observations, particularly the recognition that epigenetic changes are the mechanisms affecting several aspects of tumour cell biology, including cell growth, cell-cycle control, differentiation, DNA repair, and cell death, strongly support the rationale for reversal of methylation as an effective treatment strategy for cancer.

### Demethylating agents

In cancer cell lines, demethylating agents, such as 5-azacytidine and decitabine that inhibit DNMTs, effectively switch on expression of previously silenced genes. The greatest success achieved with demethylating agents thus far has been in the treatment of haematopoietic malignancies, particularly acute myeloid leukaemia and myelodysplasia. Both of these malignancies have been associated with hypermethylation of numerous genes, including *p15*^*INK4B*^, which in clinical studies has been reported to be aberrantly methylated in >50% patients. Analysis of a small cohort of 23 myelodysplastic syndrome (MDS) patients with hypermethylated *p15*^*INK4B*^ demonstrated a reduction in methylation following one course of low-dose 5-azacytidine and this was associated with clinical response (Daskalakis *et al*, 2002). This not only shows proof of principle, but also opens up the horizon for a number of exciting possibilities. Recent data have implicated alterations in methylation of the *p15*^*INK4B*^ gene in the transformation of MDS to acute myeloid leukaemia (Aggerholm *et al*, 2006). Reversing this epigenetic change may well reduce the risk of leukaemic transformation. Of course, despite these promising initial observations, there are several disadvantages associated with the clinical use of demethylating agents. First, demethylating agents have thus far a less impressive clinical track record in solid tumours than in haematological malignancies. This may be predominantly attributable to toxicity, although this is now felt to be due to less than optimal doses and scheduling. However, an innovative phase I study recently used demethylation agents as a ‘priming’ agent before the administration of chemotherapy with encouraging results. The combination was well tolerated, and pharmacodynamic end points of demethylation of specific target genes were met (Appleton *et al*, 2007). A significant proportion of cells in many solid tumours are non-proliferating at any given time. Demethylating agents selectively target replicating cells. As a result, this may be a limitation to the utility of the current generation of demethylating agents in non-haematological malignancies. Finally, the clinical outcome of treatment with demethylating agents may be dependant on the specific profile of methylated genes present in any individual cancer. For example, as described above, methylation of the *MGMT* gene in primary brain tumours confers sensitivity to temozolomide. Reversal of methylation in this gene would result in increased resistance to temozolomide, one of the few drugs with meaningful activity in this disease.

### HDAC inhibitors (HDACi)

Histone deacetylase inhibitors are novel agents that inhibit HDAC – the enzyme responsible for the removal of acetyl groups from specific residues on histone chains. Histone deacetylase inhibitors affect acetylation of a wide variety of proteins in cells and their precise mechanism of action as anti-tumour agents has not been definitively established. However, an attractive model proposes that HDACi affect transcription by promoting acetylation of histones – resulting in a more relaxed chromatin structure in part by chromatin remodelling and by changes in the structure of proteins in transcription factor complexes (Xu *et al*, 2007). The net result is reactivation of silenced genes in various pathways of tumour suppression. Early clinical studies showed substantial activity of HDACi in relapsed and refractory cutaneous T-cell lymphoma (Mann *et al*, 2007) at doses that caused hyperacetylation of peripheral blood mononuclear cells. Active investigation is ongoing in a variety of other tumour model systems. Combining demethylating agents with HDACi and then with cytotoxic therapy is a rational progression because epigenetic treatments could offer improved access for cytotoxic agents to the target DNA/protein complex thus allowing them to work synergistically. Numerous laboratory studies have confirmed this approach and it is making its way to the clinic.

### miRNAs

As our understanding of the function of microRNAs in tumorigenesis expands, their potential as therapeutic targets becomes ever more intriguing. Treatment of cultured cells *in vitro* with demethylating agents, either alone or in combination with HDACi, has been shown to re-activate expression of tumour suppressor miRNAs, such as miRNA-124a and mir-127, causing the corresponding repression of their oncogenic targets (Saito *et al*, 2006). To the sceptic who worries that epigenetic treatments may be too ‘blunderbluss’, miRNA-based designer therapies may prove to be the answer. Although the successful delivery of siRNAs to solid tumours has yet to be realised, designing small-molecule siRNAs to mimic tumour suppressor miRNAs could be a potential method of selectively repressing the expression of oncogenes.

## Conclusion

The next decade promises many advances in our understanding of the normal human methylome and its intricate workings, particularly in its interactions with the environment and how this may be deranged in cancer, and even more excitingly, how this may be exploited as therapy. The age in which epigenetics becomes an essential component in the clinical management of the oncology patient is getting closer.

## Figures and Tables

**Table 1:** Studies of DNA-methylation-based tests in biofluids

**Biofluid**	**Methylated genes**	**Clinical parameter**	**Reference**
Serum	*p16* ^*INK4a*^	Detection (liver)	Wong *et al* (1999)
	*GSTP1*	Detection (prostate)	Goessl *et al* (2000)
	Multiple	Detection (head and neck)	Sanchez-Cespedes *et al* (2000)
	*p16* ^*INK4a*^	Detection (oesophagus)	Hibi *et al* (2001)
	Multiple	Detection (gastric)	Lee *et al* (2002)
	*hMLH1*	Chemosensitivity (ovarian)	Gifford *et al* (2004)
	*NGFR*	Detection (colorectal)	Lofton-Day *et al* (2008)
	*SEPT9*	Detection (colorectal)	Lofton-Day *et al* (2008)
	*TMEFF2*	Detection (colorectal)	Lofton-Day *et al* (2008)
Sputum	*p16* ^*INK4a*^	Detection (lung)	Belinsky *et al* (2002)
	*hMLH1, hMSH2*	Detection (lung)	Wang *et al* (2003)
Urine	*GSTP1*	Detection (prostate)	Goessl *et al* (2000)
	*GSTP1*	Detection (prostate)	Cairns *et al* (2001)
	Multiple	Detection (renal)	Hoque *et al* (2004)
	Multiple	Detection (prostate)	Vener *et al* (2008)
Faeces	*SFRP2*	Detection (colorectal)	Müller *et al* (2004)
	Vimentin	Detection (colorectal)	Chen *et al* (2005)
Semen	*GSTP1*	Detection (prostate)	Goessl *et al* (2000)
Saliva	Multiple	Detection (head and neck)	Carvalho *et al* (2008)
Blood cells	*GSTP1*	Detection (prostate)	Goessl *et al* (2000)
	Multiple	Breast cancer risk	Widschwendter *et al* (2008)
	*IGF2*	Colorectal cancer risk	Cui *et al* (2003)
